# Acute blockade of IL-25 in a colitis associated colon cancer model leads to increased tumor burden

**DOI:** 10.1038/srep25643

**Published:** 2016-05-11

**Authors:** Tennille D. Thelen, Ryan M. Green, Steven F. Ziegler

**Affiliations:** 1 Immunology Program, Benaroya Research Institute, Seattle, Washington, USA; 2 Department of Immunology, University of Washington School of Medicine, Seattle, Washington, USA

## Abstract

Chronic inflammation within the gastrointestinal tract results in an increased risk for developing colorectal cancer. Epithelial cytokines, including interleukin-25 (IL-25), are produced in the colon and are critical for protection from parasites, but can also be pathogenic in the context of inflammatory bowel diseases and allergy. Whether IL-25 is involved in the progression from inflammation to cancer is still largely unexplored. Using a well-established murine model for colitis-induced colon cancer; we aimed to determine the role of IL-25 in this process. We found that acute IL-25 blockade resulted in greater tumor burdens compared to isotype control treated mice. Histologically, α-IL-25 treated mice had increased colitis scores compared to mice receiving isotype control antibody, as well as decreased eosinophilia. This is the first study to explore the therapeutic potential of using an IL-25 blocking antibody during a chronic inflammatory setting. Taken together these data suggest that IL-25 plays an inhibitory role in the growth and development of colonic tumors.

Ulcerative colitis (UC) is a type of inflammatory bowel disease in which immune dysregulation promotes development of ulcerations in the lining of the colon. UC has an incidence of 1.2–20.3 cases per 100,000 people per year and a prevalence of 7.6–246.0 cases per 100,000 people per year^1^. Chronic inflammation observed in patients with UC is associated with an increased risk for colorectal cancer (CRC) by as much as 2.4-fold^2^. Despite the clear correlation between UC and CRC, the immunological factors contributing to the progression from inflammation to cancer are largely unknown.

Mucosal inflammation in UC is characterized as an atypical type-2 response. IL-25, a member of the IL-17 cytokine family (IL-17E), initiates production of type-2 cytokines (IL-4, IL-5, and IL-13) thereby enhancing a T_H_2 response^3^,^4^,^5^,^6^. Through its ability to induce IgE production and eosinophilia, IL-25 plays an essential role in host defense to helminth infections. However, its production can also result in a pathogenic role in allergic disorders. IL-25 is expressed in both the colon and specific macrophage and epithelial cells located in the gut^3^,^7^, is detected in the colons of mice under steady-state conditions, and is significantly elevated upon acute exposure to a DSS-induced colitis model^8^.

While little is known about how IL-25 modulates tumor pathogenesis, recent studies have revealed it could prove to be an important therapeutic target. IL-25 was shown to induce cell death in breast cancer cells, whereas non-malignant cells were left unaffected^9^. This effect was hypothesized to be due to the increased levels of IL-25 receptor (IL-17RB) expressed in malignant tumor cells, which correlates with previous findings associating low levels of IL-17RB with aggressive breast cancers and decreased overall survival^10^,^11^. Along with breast cancers, IL-25 was shown to exert antitumor effects in melanoma, lung, colon, and pancreatic cancers with a dependence on both B cells and increased levels of IL-5 inducing eosinophilia^12^. IL-25 influences both innate and adaptive immunity to induce type-2 inflammation; because a Th2 phenotype is generally associated with tolerance in the setting of tumors, it will be critical to define the functional significance of this important cytokine in the context of tumor immunity.

There are conflicting reports on the ability of IL-25 to be either protective or detrimental in models of ulcerative colitis. In the United States, 1–1.3 million people suffer from inflammatory bowel disease^13^; given the development of new therapeutic strategies for inhibiting chronic inflammation in the gastrointestinal tract, we sought to establish the role of IL-25 in the context of colitis-driven colon cancer. We hypothesized the increased IL-25 in colitis, resulting in type-2 inflammation, would create an environment more favorable for tumor growth and development. To test this hypothesis, WT mice were treated with an α-IL-25 blocking antibody in a model of colitis-induced colon cancer. Contrary to our hypothesis, antibody suppression of IL-25 resulted in increased tumor burden compared to controls. Mice with increased tumor burden also exhibited an increased overall colitis score and decreased eosinophil infiltrates in colonic tissue. Interestingly, genetic ablation of IL-25 had no effect on tumor growth. These data suggest that while IL-25 may be promoting the type-2 inflammation associated with UC, it is also inhibiting the pro-tumorigenic potential associated with long term inflammation.

## Results

### IL-25 neutralizing antibody increases Tumor Burden in a murine Colitis-Associated Cancer (CAC) Model

**Figure 1:**
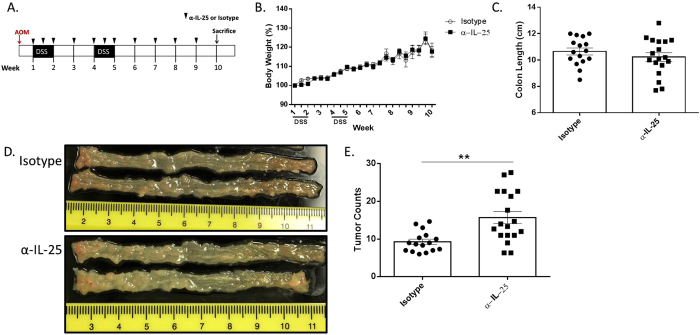
IL-25 neutralizing antibody increases tumor burden. Balb/cJ mice were given an i.p. injection of AOM on day 0, 2% DSS in drinking water for 7 days starting on days 7 and 28, and euthanized on day 70. (**A**) WT Balb/c mice were given i.p. injections of either α-IL-25 or isotype control antibody on days 7, 10, 14, 21, 28, 31, 35, 42, 49, 56, and 64. **(B)** Body weight was monitored from initial dose of DSS and expressed as percentage of initial weight. **(C)** Length of colon at euthanasia. **(D)** Colon was removed, cut lengthwise, washed with PBS, and digitally photographed. **(E)** Tumor counts based on gross analysis. Results are presented as mean ± SEM and are combined from four independent experiments with 3–5 mice per group. **P < 0.005.

To investigate the role of IL-25 in colitis-induced colon cancer, a previously established model^14^,^15^ was utilized in which the mice were treated with the carcinogen azoxymethane (AOM) followed by two rounds of 2% dextran sodium sulphate (DSS) in their drinking water. Previous studies have shown that BALB/cJ mice would develop 4 to 12 colonic neoplasms per mouse after 10 weeks. To establish what role IL-25 plays in the progression of colitis into cancer, the mice were given i.p. injections with either α-IL-25 or an isotype control starting with the initial dose of DSS, 3 days later, and weekly thereafter (Fig. 1A).

Mice were monitored and weighed 2–3 times per week for the duration of the experiment with similar weight patterns observed between α-IL-25 treated and control groups (Fig. 1B). At week 10, the mice were euthanized and the colon was removed for further analysis. DSS induced colitis is associated with a shortening of the colon, however the length of the colons from the two groups was comparable (10.66 ± 0.34 N = 12 in control group versus 10.27 ± 0.43 N = 14 in α-IL-25) (Fig. 1C).

Upon removal, the colons were cut lengthwise, and tumors were visualized using digital photography. Macroscopically, colonic tumors were observed in all mice with a higher concentration toward the distal rectum, which was expected as this is the area known to endure the greatest injury with DSS treatments. A moderate number of tumors were observed in the mid-region, though none were detected in the proximal colon of either group (Fig. 1D). Interestingly, there was greater overall tumor burden in the colons of mice treated with the α-IL-25 antibody (16.79 ± 1.95 N = 14 versus 9.61 ± 0.87 N = 12 in control group, p = 0.004) (Fig. 1D,E), suggesting that IL-25 is limiting the growth and development of colonic tumors. To understand how IL-25 might be regulating this process, further evaluation of the colonic tissue was executed.

### Increased colitis and decreased eosinophils in colons from α-IL-25 treated mice

**Figure 2:**
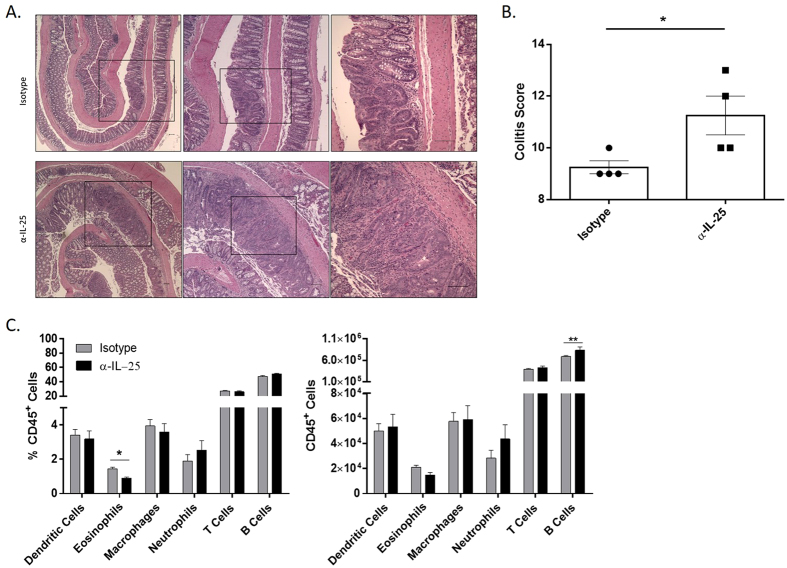
Increased colitis and decreased eosinophils in CAC mice treated with IL-25 neutralizing antibody. (**A**) H&E stained histology sections of colons from isotype (top) and α-IL-25 (bottom). Black boxes represent successive higher magnification from left to right with the black bar representing 100 μm. **(B)** Histological colon sections analyzed for colitis severity. **(C)** Single cell suspensions were made from colon tissue and labeled with fluorescently labeled antibodies to distinguish dendritic cells, eosinophils, macrophages, neutrophils, T and B cells and were analyzed by flow cytometry (percentage on left, absolute number on right). Results are presented as mean ± SEM and are representative from three independent experiments with 3–5 mice per group. *P < 0.05, **P < 0.005.

To examine the extent of disease in the colon, sections were obtained from the colons of tumor-bearing mice and stained with H&E (Fig. 2A). Colon sections underwent histological assessment for intestinal tumors as well as overall inflammation and assigned a combined colitis score^16^. Mice receiving α-IL-25 throughout the course of the CAC model had a significantly increased colitis score (11.25 ± 0.75, N = 4) compared to mice receiving isotype control antibody (9.25 ± 0.25, N = 4) (p = 0.0447) (Fig. 2B).

To determine which cell types contribute to increased tumor incidence in mice treated with α-IL-25, single cell suspensions from colonic tissue sections were stained and analyzed by flow cytometry. CD45^+^ white blood cells were gated based on surface markers to determine frequencies of dendritic cells, eosinophils, macrophages, neutrophils, T cells, and B cells. While the majority of these cell types were nearly equivalent, there was a significant decrease in the percentage of eosinophils found in mice treated with α-IL-25 compared to control mice (0.9% and 1.45% respectively, p = 0.00098) (Fig. 2C). These results suggest blockade of IL-25-mediated migration of eosinophils into tissues may be partially responsible for the increased tumor burden in these mice. To determine whether IL-25 blockade was also able to exert its effects at the genetic level, mRNA levels were established for various inflammatory cytokines as well as tumor-associated genes.

### Gene expression in CAC mice treated with α-IL-25 antibody

**Figure 3:**
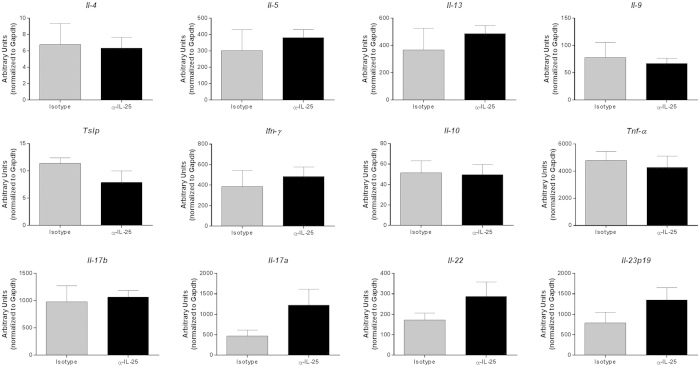
Gene expression in CAC mice treated with IL-25 neutralizing antibody. Real-time PCR analysis for various inflammatory cytokines in colon sections from AOM/DSS treated mice, expressed as arbitrary units relative to housekeeping gene. Results are presented as mean ± SEM and are representative of three independent experiments with 3–5 mice per group.

IL-25 is able to modulate the inflammatory environment by downregulating IL-17A production via IL-23^17^, as well as promote Th2 responses and reduce interferon gamma (IFN-γ) during helminth infections^18^,^19^. Mice deficient in IL-25 are highly susceptible to experimental autoimmune encephalomyelitis (EAE) with increases in numbers of IL-17, IFN-γ, and tumor necrosis factor-alpha (TNF-α) producing cells that invade the central nervous system^20^. Studies have found reduced T_H_17 cells and increased T_H_9 cells in response to IL-25^17^,^21^. To determine whether cytokine expression was affected and contributed to tumor outcome in mice treated with the IL-25 blocking antibody, a section of the distal colon was processed for real-time PCR to measure levels of gene expression for various inflammatory cytokines. Although not statistically significant, there was a trend towards increased expression levels in the Th17 pathway cytokines, *il-17a, il-22*, and *il-23p19*, in anti-IL-25 treated mice compared to controls (Fig. 3). This correlates with the previously established ability of IL-25 to down-regulate Th17 cytokines as mentioned above, and is consistent with the recently observed role for the IL-23 pathway in colon cancer^22^,^23^.

**Figure 4:**
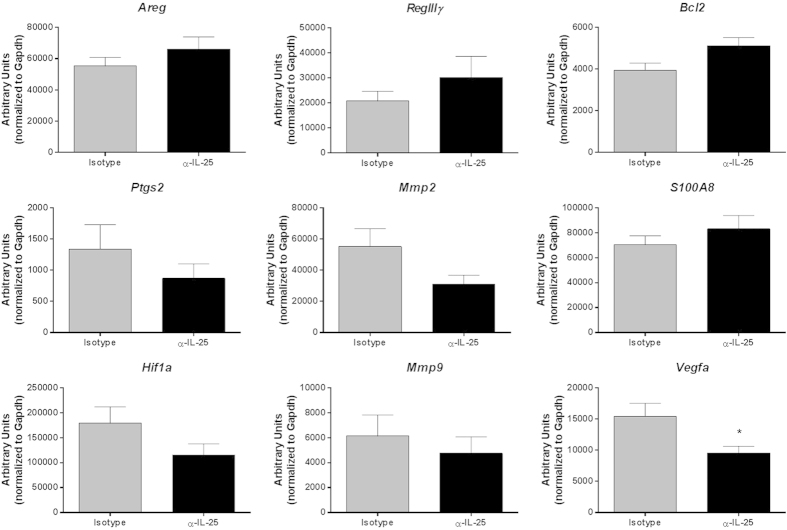
Gene expression in CAC mice treated with IL-25 neutralizing antibody. Real-time PCR analysis for tumor related genes in colon sections from AOM/DSS treated mice, expressed as arbitrary units relative to housekeeping gene. Results are presented as mean ± SEM, with asterisks representing statistical significance relative to isotype treated mice. Data are representative from three independent experiments with 3–5 mice per group.

It is unknown what effect IL-25 has on tumor-associated gene expression levels during colonic tumor development. Therefore, mRNA levels of tumor-associated genes known to be important in the AOM/DSS model were assessed to establish whether IL-25 is capable of regulating genes involved in tumor development. Genes involved in colonic epithelial neoplasia include; amphiregulin (*Areg*) (epidermal growth factor (EGF) receptor ligand and mediator of proliferation), hypoxia-inducible factor 1-alpha (*Hif1a*) (regulator of cellular and developmental response to hypoxia), prostaglandin-endoperoxide synthase 2 (*Ptges2*) (modulation of apoptosis, angiogenesis, and tumor invasiveness), vascular endothelial growth factor A (*Vegfa*) (increases vascular permeability, angiogenesis, cell migration and inhibition of apoptosis), matrix metalloproteinase-2 (*Mmp2*) and matrix metalloproteinase-9 (*Mmp9*) (involved in invasion and angiogenesis), B-cell lymphoma 2 (*Bcl2*) (anti-apoptotic protein), regenerating islet-derived protein 3 gamma (*RegIIIγ*) and calgranulin A (*S100A8*) (antimicrobial peptides). Mice treated with the α-IL-25 antibody had similar expression levels in all these genes, with a marginally significant decrease in *Vegfa* compared to isotype treated mice (Fig. 4). These findings suggest IL-25 is unable to modulate tumor development at the genetic level.

### IL-25 deficient mice show no difference in tumor burden

**Figure 5:**
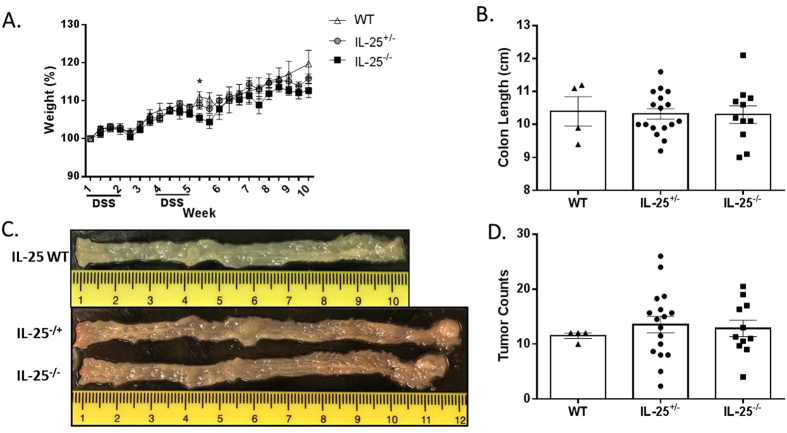
Genetic deletion of IL-25 has no effect on tumor burden. IL-25^−/−^ or littermate control mice were given an i.p. injection of AOM on day 0, 2% DSS in drinking water for 7 days starting on days 7 and 28, and euthanized on day 70. **(A)** Body weight was monitored from initial dose of DSS and expressed as percentage of initial weight. **(B)** Length of colon at euthanasia. **(C)** Colon was removed, cut lengthwise, washed with PBS, and digitally photographed. **(D)** Tumor counts based on gross analysis. Results are presented as mean ± SEM and are combined from three independent experiments.

The unexpected effect of IL-25 blockade resulting in an increase in grossly evident colonic tumors led to the hypothesis that a genetic deletion of IL-25 would also reveal an increase in tumor development. Because of the vital role gastrointestinal microbiota play in this model, it was critical to compare mice of the same parents and genetic background. To test the hypothesis, IL-25 heterozygous mice were bred in-house to obtain littermates that were wild-type, heterozygous, and full KO’s. The mice were given one i.p. injection of AOM followed by two rounds of 2% DSS as described above and monitored/weighed throughout the experiment. Interestingly, genetic deletion of IL-25 had no effect on tumor burden or other aspects of disease development (Fig. 5), suggesting that these mice are able to compensate for genetic, but not acute, IL-25 deficiency.

### Gene expression in AOM/DSS model in IL-25^−/−^ mice

**Figure 6:**
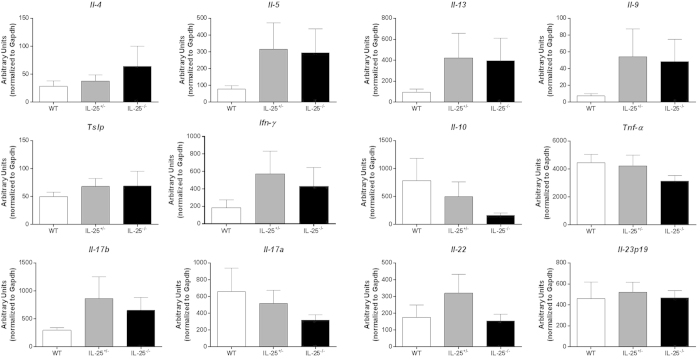
Expression of cytokine genes in AOM/DSS treated IL-25^−/−^ mice. Real-time PCR analysis for various inflammatory cytokines in colon sections from AOM/DSS treated IL-25^−/−^ or littermate control mice, expressed as arbitrary units relative to housekeeping gene. Results are presented as mean ± SEM and are combined from three independent experiments.

**Figure 7:**
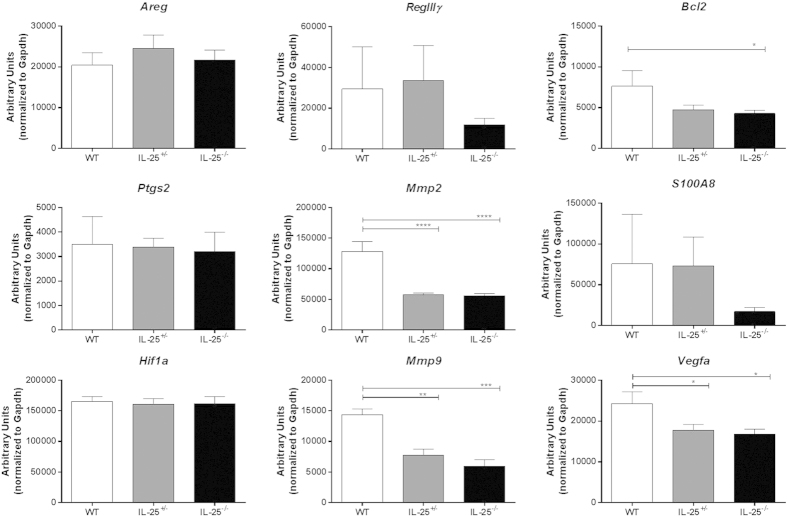
Gene expression in AOM/DSS treated IL-25^−/−^ mice. Real-time PCR analysis for tumor related genes in colon sections from AOM/DSS treated IL-25^−/−^ or littermate control mice, expressed as arbitrary units relative to housekeeping gene. Results are presented as mean ± SEM, with asterisks representing statistical significance relative to WT mice. Data are combined from three independent experiments.

As with the Balb/c mice treated with anti-IL-25 (or isotype), gene expression levels were assessed for various inflammatory cytokines using colon samples from IL-25^−/−^ mice in the AOM/DSS model. Interestingly, genetic ablation of IL-25 had no significant impact on any of the cytokine genes we measured (Fig. 6). However, there were several tumor-associated gene expression levels that were significantly reduced in the IL-25^−/−^ (and some IL-25^+/−^) mice (Fig. 7). These data suggest that, although genetic ablation does not affect tumor burden, there are changes in the gut microenvironment that appear to be IL-25 specific.

## Discussion

We have examined the role of IL-25 in an inflammation-induced colon cancer model using both antibody blockade of the cytokine and genetic deletion of signaling via IL-25 knock-out mice.

IL-25 blockade was shown to be protective in a mouse model of colitis resulting in decreased type-2 cytokines, blood eosinophils, and IgE^24^. In various models of allergic asthma, antibody-blockade of IL-25 inhibited its established ability to increase eosinophil infiltration, increase concentrations of IL-5 and IL-13, increase airway hyperresponsiveness, and induce goblet cell hyperplasia and serum IgE secretion^24^,^25^,^26^. Given the anti-inflammatory benefits discovered in both lung and gut inflammation, the use of an IL-25 blocking antibody has been suggested as a possible therapeutic treatment for human patients suffering from these types of inflammation. However, when we applied this same approach to an established murine model of colitis induced colon cancer, the tumor development surprisingly increased when mice were treated with an IL-25 blocking antibody. While colitis studies generally focus on shorter time-points post DSS treatment for evaluation, mouse colons assessed after long-term blockade of IL-25 revealed an increased colitis score compared to control mice. Our studies suggest that using an α-IL-25 antibody as a therapeutic approach could have adverse effects in colitis patients whereby it actually promotes tumorigenesis.

IL-25 injections in human tumor xenograft models including melanoma, breast, lung, colon, and pancreatic cancers were shown to have antitumor efficacy with increased eosinophils in the peripheral blood of these mice^12^. This is in alignment with the current study where we found decreased eosinophil infiltration into the colons of mice treated with an IL-25 depleting antibody. While the role of eosinophils in various tumors remains controversial, they have been found to be associated with a good prognostic indicator in gastrointestinal cancers^27^,^28^,^29^.

Our evaluation of mRNA expression in the colons of mice treated with α-IL-25 showed a trend toward increased inflammatory cytokines in the Th17 pathway (*il-17a, il-22, and il-23p19*). Previous studies in animal models of colorectal carcinoma have established Th17 cells, IL-22, and IL-23 as important drivers of inflammation leading to colonic tumor formation. Furthermore, blockade of this pathway could decrease tumorigenesis^22^,^30^,^31^. In humans, colorectal tumor samples showed increased IL-17 producing cells compared to non-tumor regions^23^. While our study showed a slight decrease in *Vegfa* expression in α-IL-25 treated mice, Liu *et al*. identified IL-17 induction of VEGF production by tumor cells as the main pro-angiogenic factor responsible for the development and progression of colorectal carcinoma. The high expression levels of *Vegfa* in both isotype and α-IL-25 treated mice indicate it likely plays a role in this model, however the mechanism of whether IL-25 is able to directly or indirectly affect its expression requires further investigation. Overall, it is possible that IL-25 blockade is allowing for increased Th17 inflammation which is promoting tumor development in these mice.

Within the gastrointestinal tract, IL-25 is an important regulator of type-2 immunity. Owyang *et al*. used IL-25^−/−^ mice to establish IL-25 as a critical factor in protection during *Trichuris* infections not only by inducing a type-2 response but also in its ability to dampen IFN-γ and IL-17 expression^19^, demonstrating an additional ability for IL-25 to limit pathologic inflammation within the gastrointestinal tract. Consistent with this, when IL-25^−/−^ mice were used in another model of helminth infection, *Nippostrongylus brasiliensis*, the protective role of IL-25 was dependent on its ability to induce type-2 cytokine production^32^. Models of colitis have shown IL-25 to be either protective^33^ or promoting^34^ of inflammation in the gastrointestinal tract. However, when IL-25^−/−^ mice were used in the colitis associated colon cancer model, we found no difference in the ultimate outcome. We speculate that while the acute blockade of IL-25 is able to impact the inflammatory environment and promote tumorigenesis, in the complete ablation of this pathway other mechanisms are able to compensate and overcome the lack of IL-25 signaling. The mechanism by which this may be occurring is currently being investigated.

In Summary, due to the known role for IL-25 to augment disease pathogenesis in UC and because UC often progresses into colon cancer, an AOM/DSS-induced murine model of colon carcinogenesis was used to establish the role for IL-25 in this environment. This is the first study of its kind to specifically examine the influence of IL-25 in a long-term inflammation-induced cancer model. We show herein that mice treated with an IL-25 blocking antibody developed greater tumor burdens and increased pathology than isotype treated control mice. These results demonstrate that the use of a blocking antibody against IL-25, while potentially therapeutic in the context of UC, should be approached with caution as it may prove to be detrimental if used long term and could actually promote tumor development.

## Materials and methods

### Ethics approval

All animal experiments in this study were approved by the Institutional Animal Care and Use Committee of Benaroya Research Institute, and were performed in accordance with the approved guidelines for animal experimentation at the Benaroya Research Institute.

### Animals

BALB/cJ mice were originally purchased from Charles River Laboratory. IL-25 deficient mice were a generous gift from Dr. Andrew N.J. McKenzie^32^. These mice had been backcrossed for 10 generations onto the Balb/cJ background prior to receiving them into our facility. All mice were bred and maintained in the Benaroya Research Institute Animal Facility under specific pathogen-free conditions.

### Colitis induced colon cancer

Mice received one intraperitoneal (i.p.) injection of azoxymethane (AOM) (10 mg/kg) on day 0, mice were given two rounds of a 2% DSS solution in their drinking water for 7 days starting on day 7 and day 28. Mice were monitored/weighed throughout the experiment and sacrificed at day 70. Colons were removed, cut lengthwise and rinsed with cold PBS. Digital photographs were taken for tumor counting, after which sections were collected for further analysis. Dextran Sodium Sulfate (36,000–50,000) MP grade was purchased from MP Biomedicals (Santa Ana, CA). AOM was purchased from Sigma-Aldrich (St Louis, MO).

### Antibody treatment

BALB/c mice were given 500 μg α-IL-25 (Amgen) via i.p. injection on days 7, 10, 14, 17, 21, 28, 35, 42, 49, 56, and 63 of colitis induced colon cancer model. Control animals were given an equivalent dose of isotype control antibody (Sigma-Aldrich). Anti-IL-25 was a gift from Amgen (Thousand Oaks, CA).

### Histology

Using the Swiss-roll technique, colon tissue was placed in 10% neutral buffered formalin (Fisher BioTech), fixed at room temperature overnight, and embedded in paraffin^35^. Paraffin blocks were sectioned and stained with H&E.

### Cell isolation and Flow cytometry

Colon tissue was cut into small pieces and digested with liberase (50 μg/mL) (Roche) and DNase (2 units/mL) (Sigma-Aldrich) in RPMI 1640 (Sigma-Aldrich) for 30 min followed by filtration through a 20 μm filter. Single cell suspensions were stained with fluorescently labeled antibodies fixed in 2% paraformaldehyde and analyzed using the BD LSRII. Antibodies used for FACS analysis (BD Biosciences and eBioscience); anti-CD45.2, anti-CD4, anti-CD16/32, anti-SiglecF, anti-MHCII, anti-CD11c, anti-CD11b, anti-CD8, anti-Ly6C, anti-Ly6G, and anti-F4/80.

### Quantification of mRNA levels by real-time PCR

Total RNA was isolated from frozen colon tissue using NucleoSpin RNA kit (TaKaRa Bio) following the manufacturer’s instructions. cDNA was synthesized using PrimeScript First Strand cDNA Synthesis Kit (TaKaRa Bio) following the manufacturer’s instructions. Genes amplified using SYBR® Premix Ex Taq^TM^, ROX Plus (Takara Bio). Real-time PCR analysis was performed on the Applied Biosystems Prism 7900HT Fast RT-PCR System. Samples were performed in triplicate and relative expression of each gene to *Gapdh* gene expression was determined by SDS 2.2.3 (Applied Biosystems) and calculated using ΔCT method. The following primer pairs were used to determine gene expression in mouse colon tissue with SYBR green; *Mmp2*, 5′-CACCACCGAGGACTATGACC-3′ and 5′-TCCTTGGTCAGGACAGAAGC-3′; *Mmp9*, 5′-TTCGACACTGACAAGAAGTGG-3′ and 5′-CCACGACCATACAGATACTGG-3′; *Areg*, 5′-GACTCACAGCGAGGATGACA-3′ and 5′-GGCTTGGCAATGATTCAACT-3; *Vegfa*, 5′-TTACTGCTGTACCTCCACC-3′ and 5′-ACAGGACGGCTTGAAGATG-3′; *Hif1a*, 5′-CGACACCATCATCTCTCTGG-3′and 5′-TGATTCAGTGCAGGATCAGC-3′; and *Ptgs2* (Cox2), 5′-CAGTCAGGACTCTGCTCACG-3′ and 5′-TTGACATGGATTGGAACAGC-3′; *Tnf-α*, 5′-CCCCAAAGGGATGAGAAGTTC-3′ and 5′-TGTGAGGGTCTGGGCCATAG-3′; *Ifn-γ*, 5′-CCTGCGCCTAGCTCTGAG-3′ and 5′-GCCATGAGGAAGAGCTGCA-3′; *il-10,* 5′-CCTCAGGATGCGGCTGAG-3′ and 5′-GCTCCACTGCCTTGCTCTTATT-3′; *il-4*, 5′-TCATCGGCATTTTGAACGAG-3′ and 5′-TTTGGCACATCCATCTCCG-3′; *il-5*, 5′-TGCCTGGAGCAGCTGGAT-3′ and 5′-TGGCTGGCTCTCATTCACACT-3′; *il-13*, 5′-ATTCCCTGACCAACATCTCCA A-3′ and 5′-CGGTTACAGAGGCCATGCAA-3′; *il-17a*, 5′-ATCAGGACGCGCAAACATGAGT-3′ and 5′-ACGCTGAGCTTTGAGGGATGAT-3′; *il-23p19*, 5′-ATCACCCCCGGGAGACCCAA-3′ and 5′-TGCTGCTCCGTGGGCAAAGAC-3′; *tslp*, 5′-AGGCTACCCTGAAACTGAGA-3′ and 3′-GGAGATTGCATGA|AGGAATAC-5′. Primer pairs and probes using TaqMan Gene Expression Assays (Life Technologies) for mouse; *Gapdh* (Mm99999915_g1)*, il-9* (Mm01235642_g1)*, Bcl2* (Mm00477631_m1), *Reg3γ* (Mm01181783_g1), and *S100A8* (Mm00496696_g1).

### Statistics

All statistical analyses were performed using GraphPad Prism software verson 6.0. Results are expressed as means ± SEM. Statistical test used to analyze data sets between two groups was the Student t test and a one-way ANOVA to compare difference between three or more groups. P values of <0.05 were considered statistically significant.

## Additional Information

**How to cite this article**: Thelen, T. D. *et al*. Acute blockade of IL-25 in a colitis associated colon cancer model leads to increased tumor burden. *Sci. Rep.* **6**, 25643; doi: 10.1038/srep25643 (2016).
